# Increasing Incidence of Invasive Group A *Streptococcus* Disease, Idaho, USA, 2008–2019

**DOI:** 10.3201/eid2809.212129

**Published:** 2022-09

**Authors:** Eileen M. Dunne, Scott Hutton, Erin Peterson, Anna J. Blackstock, Christine G. Hahn, Kathryn Turner, Kris K. Carter

**Affiliations:** Centers for Disease Control and Prevention, Atlanta, Georgia, USA (E.M. Dunne, A.J. Blackstock, K.K. Carter);; Idaho Department of Health and Welfare, Boise, Idaho, USA (E.M. Dunne, S. Hutton, E. Peterson, C.G. Hahn, K. Turner, K.K. Carter)

**Keywords:** group A Streptococcus, Streptococcus pyogenes, strep A, bacteria, bacterial infection, case-case study, streptococcal disease, Idaho, United States, streptococci

## Abstract

We investigated invasive group A *Streptococcus* epidemiology in Idaho, USA, during 2008–2019 using surveillance data, medical record review, and *emm* (M protein gene) typing results. Incidence increased from 1.04 to 4.76 cases/100,000 persons during 2008–2019. *emm* 1, 12, 28, 11, and 4 were the most common types, and 2 outbreaks were identified. We examined changes in distribution of clinical syndrome, patient demographics, and risk factors by comparing 2008–2013 baseline with 2014–2019 data. Incidence was higher among all age groups during 2014–2019. Streptococcal toxic shock syndrome increased from 0% to 6.4% of cases (p = 0.02). We identified no differences in distribution of demographic or risk factors between periods. Results indicated that invasive group A *Streptococcus* is increasing among the general population of Idaho. Ongoing surveillance of state-level invasive group A *Streptococcus* cases could help identify outbreaks, track regional trends in incidence, and monitor circulating *emm* types.

Invasive group A *Streptococcus* (iGAS) disease is a severe bacterial infection caused by *Streptococcus pyogenes*, sometimes called strep A; ≈25,000 cases occurred in the United States in 2019 (*1*). iGAS disease is defined as illness associated with detection of group A *Streptococcus* in a normally sterile site, such as blood, pleural fluid, joint fluid, or deep tissue. iGAS has different clinical syndromes, including bacteremia without focus, pneumonia, and cellulitis, and severe manifestations, include necrotizing fasciitis and streptococcal toxic shock syndrome (STSS). The highly variable M protein, encoded by *emm* (M protein gene), is essential for virulence and serves as the primary target for epidemiologic typing of GAS (*2*). Most patients with iGAS are hospitalized and the estimated case-fatality rate in the United States is 12% (*3*). Numerous risk factors are associated with iGAS, including older age, diabetes, HIV infection, heart disease, cancer, obesity, injection drug use, long-term care facility (LTCF) residence, homelessness, preceding or concurrent influenza infection, and exposure to children with sore throats (*4**–**9*). No licensed vaccines for GAS exist despite its substantial global disease burden: an estimated 1.78 million new cases of severe GAS (iGAS, acute rheumatic fever, rheumatic heart disease, and poststreptococcal glomerulonephritis) and 517,000 deaths occurring each year (*10*). In 2019, the World Health Organization recognized GAS as a leading cause of infectious disease burden and proposed better characterization of GAS epidemiology as a priority activity for vaccine development (*11*).

During the past decade, iGAS incidence has increased in the United States, Canada, Ireland, Australia, and New Zealand (*1*,*12**–**16*). Reasons for these increases are unclear but could include changing demographics (e.g., an aging population at higher risk for iGAS), rising prevalence of other risk factors associated with iGAS infection, or changes in circulating *emm* types. In some US states, iGAS outbreaks among specific populations (e.g., persons experiencing homelessness or who inject drugs) or the emergence of rarely occurring *emm* types were linked to increases in iGAS incidence (*17**–**21*). In Idaho, a rural, western US state with a 2020 population of ≈1.8 million persons, iGAS is a reportable disease. We conducted a retrospective analytical study using surveillance data, *emm* typing results, and medical record review to describe the epidemiology of iGAS in Idaho during 2008–2019. In addition, we compared cases reported during 2014–2019 with cases from a lower-incidence 2008–2013 baseline period to determine whether clinical syndromes, patient demographics, and risk factors were associated with increased iGAS incidence.

## Methods

### Case Identification and Data Collection

We defined confirmed iGAS cases as *S. pyogenes* isolated by culture from a normally sterile site (e.g., blood, or cerebrospinal, joint, pleural, or pericardial fluids). Cases of iGAS in Idaho must be reported to the Idaho Division of Public Health or local public health agencies, according to Idaho reportable disease rules (https://publicdocuments.dhw.idaho.gov/WebLink/DocView.aspx?id=6798&dbid=0&repo=PUBLIC-DOCUMENTS). Reportable disease surveillance is a passive surveillance system, and with rare exceptions, reports are submitted by laboratories. After iGAS cases are reported, local public health district epidemiologists collect data and determine whether a given case is associated with a congregate setting or an outbreak. For this retrospective study, we used information about iGAS cases reported during 2008–2019, including patient demographics, laboratory reports, and data obtained from public health investigations, recorded in the Idaho National Electronic Disease Surveillance System Base System (NBS) and from review of medical records to collect additional clinical and risk factor information. We viewed medical records in the Idaho Health Data Exchange or obtained them by faxing medical record requests to hospitals. Because reported iGAS cases increased substantially from 2013 to 2014, we reviewed reporting facilities and submission procedures to determine whether potential changes in reporting methods (for example, an increase in electronic over manual reporting) might have contributed to the increase in reported cases.

We obtained data concerning hospitalization and death from NBS or medical records; however, the records for fatal cases did not always indicate whether iGAS was considered a cause of or contributing factor to death. Case information encompassed patient demographics (i.e., age, sex, race and ethnicity), type of residence, clinical syndrome (i.e., pneumonia, bacteremia, meningitis, cellulitis), and underlying conditions and behavioral risk factors (i.e., diabetes, skin wound or injury, cancer, chronic obstructive pulmonary disease or emphysema, obesity, dialysis or renal failure, immunosuppression, postpartum status, recent surgery, alcohol abuse, injection drug use, current smoking). We selected variables for data abstraction based on the Centers for Disease Control and Prevention (CDC) Active Bacterial Core surveillance (ABCs) case report form (https://www.cdc.gov/abcs/downloads/abcs-case-rpt-form.pdf). We collected data on other risk factors (e.g., GAS pharyngitis, recent or concurrent influenza infection, household member with GAS infection), when available, from medical records or NBS.

Except for results from 2 isolates from 2012 that underwent *emm* typing at the Boise Veterans Administration Medical Center, *emm* typing results were provided by the Idaho Bureau of Laboratories, which began *emm* typing GAS isolates voluntarily submitted by diagnostic laboratories in 2014. *emm* typing at Idaho Bureau of Laboratories was conducted by PCR amplification and Sanger sequencing of the *emm* gene according to CDC protocols (https://www.cdc.gov/streplab/protocol-emm-type.html). *emm* types were assigned using a CDC M type–specific sequence database library within the bionumerics software platform (ftp://ftp.cdc.gov/pub/infectious_diseases/biotech/tsemm). Sequences were verified using the CDC Blast-*emm* database (https://www2.cdc.gov/vaccines/biotech/strepblast.asp).

### Data Analysis

We used Stata software version 14.2 (StataCorp, LLC, https://www.stata.com) to conduct data analysis and compiled categorical data as percentages and continuous data as medians with interquartile ranges (IQRs). We calculated annual incidence per 100,000 persons overall and by age group using annual census data for Idaho population estimates (https://www.census.gov/data.html); we age-standardized incidence rates to the average population distribution for Idaho during 2008–2019 and reported with 95% CIs. We calculated incidence estimates by race and ethnicity using data from the Idaho Bureau of Vital Records and Health Statistics (Appendix).

To investigate the increase in iGAS incidence, we conducted a case-case analysis, comparing cases from the higher incidence period (2014–2019) with cases from the lower incidence baseline period (2008–2013). We used the χ^2^ test to compare associations between periods and disease syndromes, deaths, hospitalization status, and predisposing conditions; we used the Mann-Whitney U test to compare median length of hospital stay. We used logistic regression models to compare demographics, underlying conditions, and other risk factors between the 2 periods to identify any factors that might be positively associated with the higher incidence period (Appendix). We included age group, obesity, residence type, and injection drug use as variables in the multivariable logistic regression model. We reported results as odds ratios for the univariable models and adjusted odds ratios for the multivariable model and calculated 95% CIs for all results. Odds ratios >1 indicated higher odds of cases with that risk factor being in the 2014–2019 period, compared with the baseline period. Idaho Division of Public Health and CDC determined this project to be nonresearch (public health practice). We conducted this activity consistent with applicable federal law and CDC policy.

## Results

**Figure 1:**
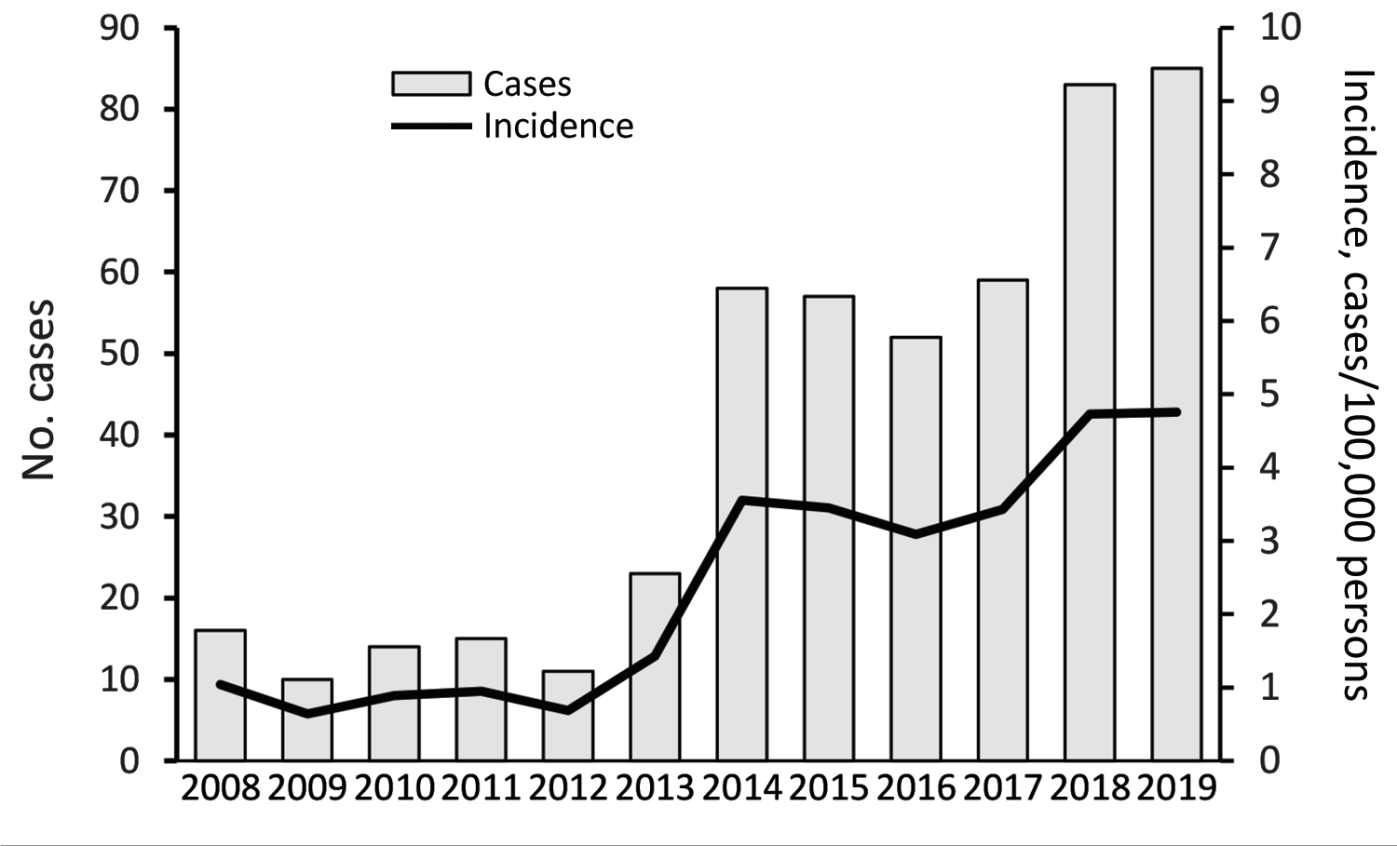
Annual number of reported cases of invasive group A *Streptococcus* (n = 483) and incidence (cases per 100,000 persons) from an investigation in Idaho, USA, comparing cases reported during 2014–2019 with cases from a lower-incidence baseline period, 2008–2013.

**Figure 2:**
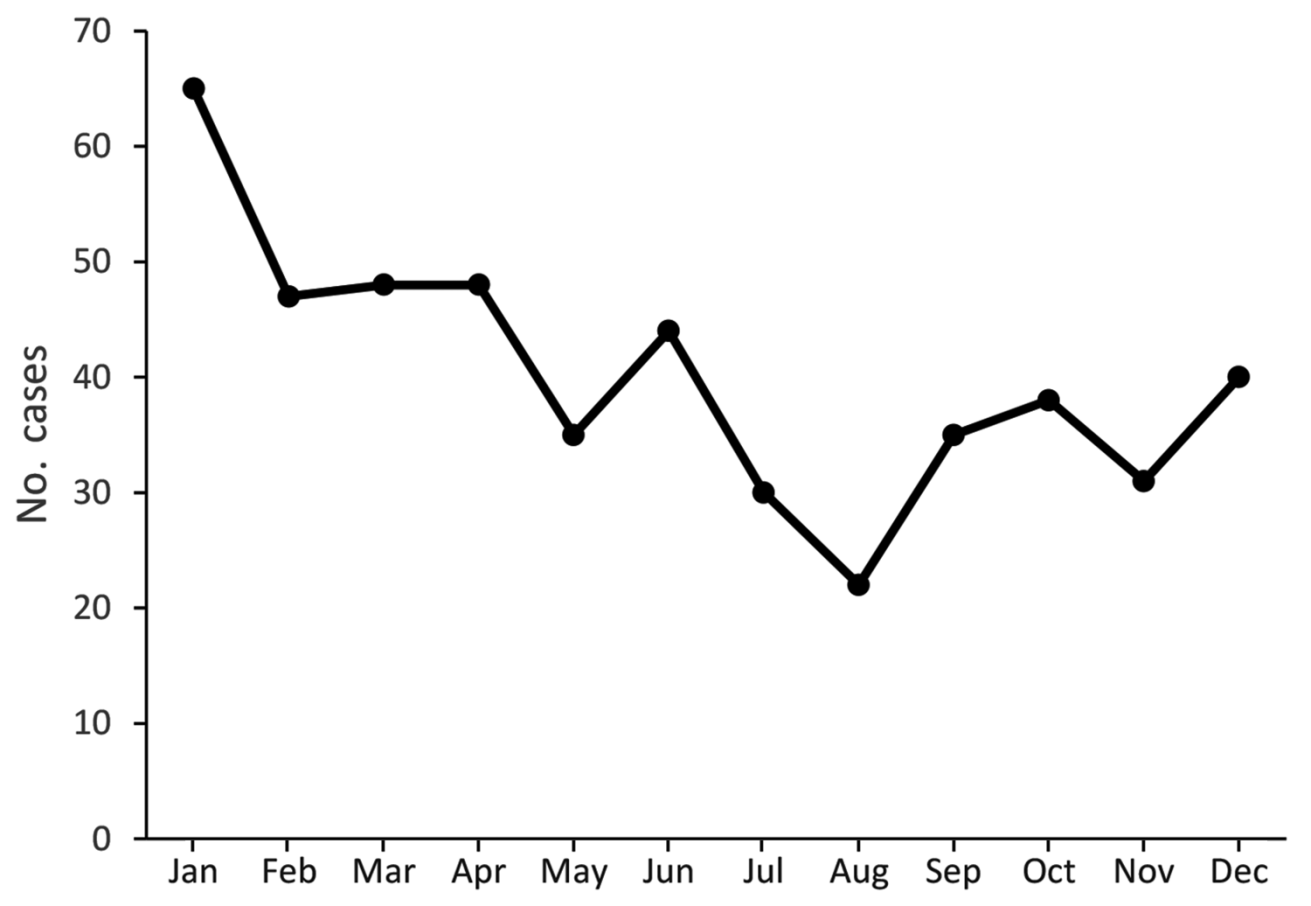
Seasonality of invasive group A *Streptococcus* (n = 483) by month of diagnosis, from an investigation in Idaho, USA, comparing cases reported during 2014–2019 with cases from a lower-incidence baseline period, 2008–2013.

During 2008–2019, a total of 483 cases of iGAS disease were reported among Idaho residents. Annual disease incidence per 100,000 persons increased from 1.04 to 4.76 during 2008–2019 (Figure 1). Case numbers were highest in January and lowest in August with similar patterns across years (Figure 2; Appendix Figure 1). We identified no changes in the surveillance system that might have led to increased case reporting. Overall, 52.8% of cases occurred among men; median age of case-patients was 62 years (Table 1). Data concerning race and ethnicity were available for 444 (91.9%) patients, most of whom were white, non-Hispanic persons (87.8%), followed by Hispanic (5.0%) and American Indian or Alaska Native persons (4.1%). For comparison, the 2018 population of Idaho was estimated to be 83.1% white non-Hispanic, 12.7% Hispanic, and 2.0% American Indian or Alaska Native (*22*). The average annual incidence per 100,000 persons was 2.39 during 2008–2019; incidence was 2.55 among white non-Hispanic, 1.03 among Hispanic, and 5.19 among American Indian and Alaska Native persons. Data concerning residence type were available for 428 (88.6%) patients; most (349, 81.5%) resided in a private residence, followed by 63 (14.7%) in a LTCF or nursing home.

**Table 1:** Demographics of patients with invasive group A *Streptococcus* disease, overall and by 6-year periods, Idaho, USA, 2008–2019*

Variable	Overall, N = 483	2008–2013, n = 89	2014–2019, n = 394
Sex			
F	228 (47.2)	48 (53.9)	180 (45.7)
M	255 (52.8)	41 (46.1)	214 (54.3)
Age, y, median (interquartile range)	62 (41–75)	60 (37–75)	64 (41–75)
Age group, y			
<5	24 (5.0)	4 (4.5)	20 (5.1)
5–17	43 (4.8)	5 (5.6)	18 (4.6)
18–34	45 (9.3)	12 (13.5)	33 (8.4)
35–49	58 (12.0)	10 (11.2)	48 (12.2)
50–64	104 (21.5)	20 (22.5)	84 (21.3)
65–79	147 (30.4)	21 (23.6)	126 (32.0)
≥80	92 (17.0)	17 (19.1)	65 (16.5)
Race/ethnicity	n = 444†	n = 83†	n = 361†
Hispanic	22 (5.0)	2 (2.4)	20 (5.5)
Non-Hispanic			
White	390 (87.8)	74 (89.2)	316 (87.5)
Black	4 (0.9)	1 (1.2)	3 (0.8)
American Indian/Alaska Native	18 (4.1)	5 (6.0)	13 (3.6)
Asian	5 (1.1)	1 (1.2)	4 (1.1)
Native Hawaiian/Pacific Islander	5 (1.1)	0 (0.0)	5 (1.4)
Residence type	n = 428*	n = 71*	n = 357*
Private residence	349 (81.5)	56 (78.9)	293 (82.1)
Long-term care or nursing facility	63 (14.7)	13 (18.3)	50 (14.1)
Homeless	9 (2.1)	1 (1.4)	8 (2.2)
Correctional facility	7 (1.6)	1 (1.4)	6 (1.7)

*Values are no. (%) patients except as indicated. †Excludes cases with missing data.

**Figure 3:**
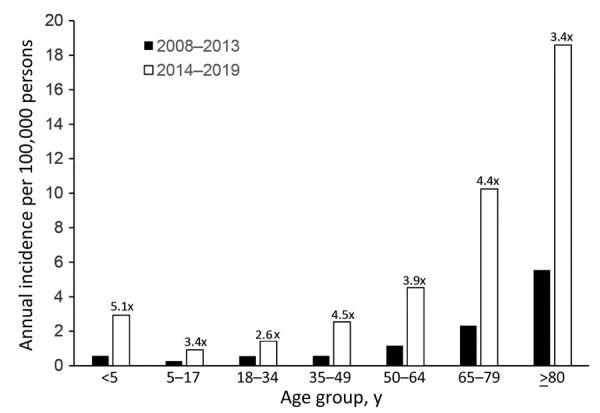
Average annual incidence of invasive group A *Streptococcus* (cases per 100,000 persons) during 2008–2013 (n = 89) and 2014–2019 (n = 394), by age group, Idaho, USA. The fold change for 2014–2019 compared with 2008–2013 is shown for each age group above the paired columns.

Mean annual incidence per 100,000 persons was 0.94 during the 2008–2013 baseline period (n = 89 cases) and 3.84 during 2014–2019 (n = 394 cases). Patient demographics were similar between the periods (Table 1); we included odds ratios in risk factor analysis (Table 2). Disease incidence increased between periods among all age groups (Figure 3). The mean annual age-standardized incidence per 100,000 persons increased from 1.0 (95% CI 0.5–1.5) during 2008–2013 to 3.7 (95% CI 2.8–4.6) for 2014–2019. iGAS incidence increased in all 7 of Idaho’s public health districts (Appendix Figure 2).

**Table 2:** Individual risk factor and multivariable analysis of risk factors comparing invasive group A *Streptococcus* cases during 2014–2019 with cases from the 2008–2013 baseline period, Idaho, USA

Variable	Unadjusted odds ratio* (95% CI)	p value	Adjusted odds ratio† (95% CI)	p value
Sex				
F	Referent			
M	1.4 (0.9–2.2)	0.16		
Age group, y				
0–17	1.2 (0.5–2.8)	0.71	1.3 (0.5–3.3)	0.63
18–49	Referent		Referent	
50–64	1.2 (0.6–2.3)	0.65	1.6 (0.7–3.8)	0.29
65–79	1.7 (0.9–3.2)	0.13	1.7 (0.8–3.8)	0.16
≥80	1.1 (0.5–2.1)	0.90	1.1 (0.5–2.6)	0.84
Ethnicity				
Non-Hispanic	Referent			
Hispanic	2.4 (0.5–10.4)	0.25		
Residence type				
Private	Referent		Referent	
Long-term care or nursing facility	0.7 (0.4–1.4)	0.37	0.7 (0.3–1.5)	0.33
Correctional facility	1.2 (0.1–9.7)	0.90	1.9 (0.1–36.7)	0.67
Homeless	1.5 (0.2–12.5)	0.69	0.7 (0.1–4.8)	0.74
Underlying conditions				
Diabetes	0.9 (0.5–1.5)	0.68		
Heart disease: congestive heart failure or coronary artery disease	1.3 (0.7–2.3)	0.46		
Obesity	1.3 (0.7–2.4)	0.49	1.2 (0.6–2.5)	0.58
Chronic kidney disease or failure	1.6 (0.7–3.6)	0.30		
Chronic obstructive pulmonary disease	0.8 (0.4–1.8)	0.62		
Cancer	2.4 (0.7–7.9)	0.17		
Immunosuppression	2.0 (0.5–8.6)	0.37		
Hepatitis C or chronic liver disease	1.5 (0.4–6.9)	0.57		
Other‡	2.3 (0.3–18.2)	0.42		
Any underlying condition	0.9 (0.5–1.7)	0.75		
Other risk factors				
Skin injury	1.0 (0.6–1.7)	0.97		
Cigarette smoking	0.9 (0.5–1.9)	0.82		
Alcohol abuse	0.9 (0.3–2.3)	0.75		
Injection drug use	3.4 (0.2–60.0)	0.40	3.2 (0.2–63.0)	0.45

*Standard logistic regression analysis performed unless otherwise noted. An odds ratio >1 indicates higher odds of being in the 2014–2019 period. †Firth logistic regression used to account for separation attributable to limited sample size and highly predictive risk factors. For multivariable analysis, results for residence type and injection drug use represent total effect and results for age group and obesity represent direct effect. ‡Other underlying conditions include paralysis, neurologic conditions, and developmental delay.

Medical records were available for 383/483 (79.3%) cases, 62/89 (70%) during the baseline 2008–2013 period and 321/394 (81.5%) during 2014–2019. Information concerning deaths was available for 464 (96.0%) patients, 55 (11.9%) of whom died. The case-fatality rate was 14/84 (16.7%) for the baseline period and 41/380 (10.4%) during 2014–2019 (p = 0.132). Of 471 patients with data on hospitalization status, 441 (93.6%) were hospitalized. The proportion of patients hospitalized was slightly lower during 2008–2013 (88.4%, 76/86) compared with 94.8% (365/385) during 2014–2019 (p = 0.027). Data on length of hospital stay were available for 385 hospitalized patients. Median stay was 5 days (IQR 4–9) for all hospitalized patients: 5 days (IQR 3–8) for 58 patients hospitalized during 2008–2013 compared with 6 days (IQR 4–9; p = 0.118) for 327 patients hospitalized during 2014–2019.

Cellulitis was the most common clinical syndrome, reported in 41.4% of cases, followed by bacteremia without focus (34.2%) and pneumonia (16.8%) (Table 3). STSS, a rare but severe syndrome caused by GAS infection, was identified in 25 cases, all during 2014–2019 (p = 0.02). Toxic shock syndrome is a reportable disease in Idaho; however, 11 (44%) STSS cases were identified only retrospectively through medical record review. Ages of STSS patients ranged from 10 months to 82 years; 6/22 (27%) died (data missing for 3 patients). We observed no other differences in clinical syndromes between periods. GAS was cultured from blood in 92.9% (442/476) of cases, with no differences over time: 74/82 (90.2%) for the baseline period compared with 368/394 (93.4%; p = 0.31) during 2014–2019. Data on postpartum status were available for 47/50 women 15–44 years of age, 10/47 (21%) of whom were postpartum, with no difference between periods: 2/9 (22%) during the baseline period compared with 8/38 (21%; p = 0.94) during 2014–2019.

**Table 3:** Clinical syndromes of invasive group A *Streptococcus* disease, overall and by 6-year periods, Idaho, USA, 2008–2019*

Type of infection or clinical syndrome	No. (%) patients			p value‡
	Overall, N = 476†	2008–2013, n = 82†	2014–2019, n = 394	
Bacteremia without focus§	163 (34.2)	34 (41.5)	129 (32.7)	0.13
Cellulitis	197 (41.4)	30 (36.6)	167 (42.4)	0.33
Pneumonia	80 (16.8)	15 (18.3)	65 (16.5)	0.69
Streptococcal toxic shock syndrome	25 (5.3)	0 (0.0)	25 (6.4)	0.02
Septic arthritis	24 (5.0)	6 (7.3)	18 (4.6)	0.30
Empyema	19 (4.0)	4 (4.9)	15 (3.8)	0.65
Necrotizing fasciitis	12 (2.5)	4 (4.9)	8 (2.0)	0.14
Osteomyelitis	6 (1.3)	0 (0.0)	6 (1.5)	0.26
Meningitis	4 (0.8)	0 (0.0)	4 (1.0)	0.36
Other¶	3 (0.6)	1 (1.2)	2 (0.5)	0.46

*Cases can have >1 type of infection or clinical syndrome. †Excludes 7 cases with missing data on type of infection or clinical syndrome, all during 2008–2013. ‡By χ^2^ test. §Group A *Streptococcus* isolated from blood, with no other clinical syndrome identified. ¶Other includes abscess, epiglottitis, and pelvic inflammatory disease.

**Figure 4:**
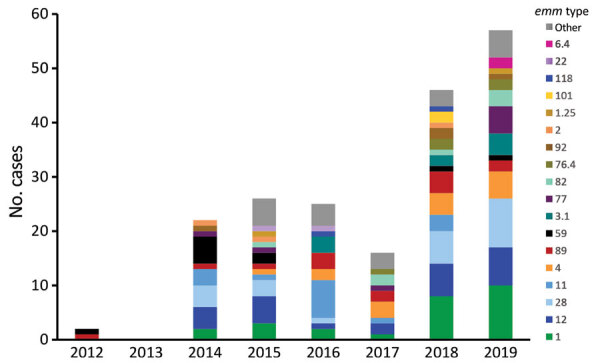
Invasive group A *Streptococcus emm* types (n = 194), Idaho, USA, 2012–2019, from an investigation in Idaho comparing invasive group A *Streptococcus* cases reported during 2014–2019 with cases from a lower-incidence baseline period, 2008–2013. The Idaho Bureau of Laboratories only began *emm* typing in 2014; 2012 data are from the Boise Veterans Administration Medical Center. Data for 2013 were unavailable.

*emm* typing was conducted on bacterial isolates from 194 (40.2%) iGAS cases, 2/89 (2.3%) during 2008–2013 and 192/394 (48.7%) during 2014–2019. In total, we identified 38 different *emm* types; the most common were types 1 (n = 26, 13%), 12 (n = 25, 13%), 28 (n = 23, 12%), 11 (n = 15, 8%), and 4 (n = 15, 8%) (Figure 4; Appendix Table 1). emm typing results were available for 14/25 (56%) STSS cases, from which 10 *emm* types were observed; types 1 (n = 3), 12 (n = 2), and 1.25 (n = 2) were identified in >1 patient.

Two outbreaks were previously identified on the basis of epidemiologic information and *emm* types. During July–September 2016, an outbreak of 5 cases of iGAS caused by *emm59* occurred among residents of a single county. During 2014–2016, an outbreak of iGAS occurred among residents of a LTCF; 9 cases were *emm11*, and 4 cases did not have *emm* typing conducted. In addition, a household cluster of 2 cases, both associated with injection drug use, occurred in May 2015, but *emm* typing was not conducted. In total, 16/394 (4.1%) of cases during 2014–2019 were associated with a cluster or outbreak.

Information concerning underlying medical conditions was available for 432 (90.2%) patients, 69/89 (78%) during 2008–2013 and 363/394 (92.1%) during 2014–2019. Of these patients, 73.8% had ≥1 underlying condition; diabetes (41.2%), heart disease (26.9%), and obesity (22.0%) were the most common (Table 4). No patients had HIV infection. Overall, 201/412 (47.8%) patients had skin injuries, and nonsurgical wounds were most frequently reported. Injection drug use was reported for 8/386 (2.1%) and methamphetamine use for 6/386 (1.6%) patients, all during the 2014–2019 period. Data for other risk factors (GAS pharyngitis, household member with GAS infection, influenza infection) were available for 389 patients, and GAS pharyngitis was identified in 8.0% of patients. In regression analyses, we observed no associations between demographic or risk factors and the higher-incidence 2014–2019 period (Table 2). Injection drug use had an adjusted odds ratio of 3.2; however, the limited number of observations yielded wide 95% CIs.

**Table 4:** Underlying conditions and risk factors of patients with invasive group A *Streptococcus* disease, overall and by 6-year periods, Idaho, 2008–2019

Risk factor	No. (%) patients		
	Overall, N = 432*	2008–2013, n = 69*	2014–2019, n = 363*
Any underlying condition			
No	113 (26.2)	17 (24.6)	96 (26.5)
Yes	319 (73.8)	52 (75.4)	267 (73.6)
Condition			
Diabetes	178 (41.2)	30 (43.5)	148 (40.8)
Heart disease: congestive heart failure or coronary artery disease	116 (26.9)	16 (23.2)	100 (27.6)
Obesity	95 (22.0)	13 (18.8)	82 (22.6)
Kidney disease: chronic kidney disease or chronic kidney failure	61 (14.1)	7 (10)	54 (14.9)
Chronic obstructive pulmonary disease or emphysema	43 (10.0)	8 (11.6)	35 (9.6)
Cancer	38 (8.8)	3 (4.3)	35 (9.6)
Immunosuppression	22 (5.1)	2 (2.9)	20 (5.5)
Hepatitis C or chronic liver disease	18 (4.2)	2 (2.9)	16 (4.4)
Other†	13 (3.0)	1 (1.4)	12 (3.3)
Total underlying conditions			
0	115 (26.6)	17 (24.6)	98 (27.0)
1	113 (26.2)	24 (34.8)	89 (24.5)
2	93 (21.5)	10 (14.5)	83 (22.9)
>3	111 (25.7)	18 (26.1)	93 (25.6)
Skin injury			
Any skin injury	n = 412*	n = 70*	n = 342*
No	211 (51.2)	36 (51.4)	175 (51.2)
Yes	201 (47.8)	34 (48.6)	167 (48.8)
Type of skin injury‡	n = 201	n = 34	n = 167
Nonsurgical wound	127 (63.2)	22 (64.7)	105 (62.9)
Surgical wound	17 (8.5)	3 (8.8)	14 (8.4)
Trauma§	47 (23.4)	8 (23.5)	39 (23.4)
Burn	2 (1.0)	0	2 (1.2)
Skin breakdown	3 (1.5)	1 (2.9)	2 (1.2)
Other	5 (2.5)	0	5 (3.0)
Behavioral risk factors			
Current cigarette smoking¶	n = 375*	n = 60*	n = 315*
No	310 (82.7)	49 (81.6)	261 (82.9)
Yes	65 (17.3)	11 (18.3)	54 (17.1)
Substance abuse	n = 386*	n = 63*	n = 323*
Alcohol abuse	27 (7.0)	5 (7.9)	22 (6.8)
Methamphetamine use	6 (1.6)	0	6 (1.8)
Injection drug use	8 (2.1)	0	8 (2.5)
None of the above	346 (89.6)	58 (92.1)	288 (89.2)
Other risk factors	n = 389*	n = 66*	n = 323*
GAS pharyngitis	31 (8.0)	7 (10.6)	24 (7.4)
Household member with GAS	8 (2.1)	2 (3.0)	6 (1.9)
Influenza	12 (3.1)	4 (6.1)	8 (2.5)
None of the above	336 (86.4)	54 (81.8)	282 (87.3)

*Excludes missing data. †Other underlying conditions include paralysis, neurologic conditions, and developmental delay. ‡Data from 201 cases with a skin injury reported. §Cut, laceration, or puncture wounds. ¶Does not include e-cigarette use or vaping.

## Discussion

Using statewide reportable disease surveillance data supplemented by information from medical record review, we investigated the epidemiology of iGAS in Idaho over a 12-year period, during which incidence increased ≈>4-fold. The ABCs program, which does not include data from Idaho, identified a similar increase in nationwide iGAS incidence per 100,000 persons, from 3.69 in 2008 to 7.63 in 2019 (*1*,*12*,*23*). In Canada, incidence per 100,000 persons rose from 4.42 in 2008 to 8.12 in 2019 (https://diseases.canada.ca/notifiable/charts).

In Idaho, average annual iGAS incidence during our study was 2-fold as high among American Indian or Alaska Native persons compared with white non-Hispanic persons. Higher iGAS incidence among indigenous compared with nonindigenous populations has been reported in multiple settings, including the United States, Australia, and New Zealand (*24*). In Alberta, Canada, iGAS incidence was 6-fold as high and increasing more rapidly among First Nation compared with non-First Nation populations, rising to 52.2 cases per 100,000 persons in 2017 (*25*). Neither the proportion of cases occurring among American Indian and Alaska Native persons nor the racial and ethnic distribution of Idaho’s population changed substantially during our study period (Appendix Table 2).

Approximately three quarters of iGAS patients had >1 underlying medical condition; diabetes, obesity, and skin injuries were common, emphasizing the need for diabetes management and wound care to reduce the risk for iGAS associated with these conditions (*4**,**5*). The high proportion of cases associated with skin injury or infection is consistent with national data; in 2019, a total of 44.7% of iGAS case-patients had cellulitis (*1*). Two outbreaks and a household cluster were identified through routine surveillance, all during 2014–2019; *emm* typing made outbreak detection easier. Because the state public health laboratory only began *emm* typing in 2014, earlier outbreaks might not have been detected. We recommend that states with available resources conduct *emm* typing to enhance surveillance and outbreak detection.

The incidence of iGAS in Idaho, 4.76 cases per 100,000 persons in 2019, is lower than the national incidence rate, 7.63 per 100,000 persons in 2019, possibly because Idaho iGAS estimates are based on passive data reporting, whereas national estimates are based on data from the ABCs active surveillance system. Other factors related to demographics or prevalence of risk factors might also contribute to lower Idaho compared with national iGAS incidence estimates. Increased iGAS rates across all age groups and in all geographic regions of Idaho during 2008–2019 present cause for concern. We identified no changes in surveillance procedures that might have led to increased case reporting. However, we did not assess potential changes in clinical practice (e.g., the number of blood cultures ordered by emergency departments), which might have affected case detection.

We identified a rise in STSS incidence: 25 cases occurred during 2014–2019, compared with none during 2008–2013, although these numbers do not account for the increase in overall iGAS cases. We found evidence of STSS underreporting, as has been reported in other studies (*26*). Cases of STSS from the baseline period might have been missed because medical records were only available for 70% of iGAS patients. We observed 10 different *emm* types among STSS cases that had an isolate submitted for typing, indicating a lack of clonality. Of note, a study in the Netherlands identified a temporal association between STSS and influenza A virus (*27*). However, STSS cases in Idaho occurred throughout the year, without seasonal patterns that might have been associated with influenza.

*emm1* was the most common *emm* type identified in Idaho and also the most common cause of iGAS nationally during 2008–2019 (*1*,*12*,*23*). The diversity of *emm* types observed in Idaho was consistent with reports from high-income countries in North America and Europe (*28**–**30*). Although no vaccines targeting GAS are available, the 30-valent M protein–based strep A vaccine, one of the few vaccines under development in phase 1 or 2 clinical trials, would potentially cover 167/194 (86.1%) *emm* types from Idaho iGAS cases, and possibly more through cross-protection within *emm* clusters based on M protein structure (*31**–**33*).

We identified no associations between risk factors and increased incidence during 2014–2019. In New Mexico, increases in iGAS incidence were linked to rises in cases among persons experiencing homelessness and persons injecting drugs (*17*). Similarly, an analysis of ABCs national surveillance data concluded that injection drug use and homelessness likely contribute to increasing iGAS incidence in the United States, noting that particular *emm* types are increasing among this patient population (*7*). Factors responsible for increased incidence of iGAS during 2003–2017 in Alberta, Canada, were not clear, and multiple risk factors likely contributed, particularly alcohol abuse and drug use (*13*). In Idaho, only 8 patients reported injection drug use and 9 experiencing homelessness. Although these conditions might be underreported, homelessness and injection drug use do not appear to be driving the increase in iGAS in Idaho, although they might be contributing factors. We found no evidence that alcohol abuse was contributing to the increase in iGAS in Idaho.

Our results suggest that iGAS is increasing among the general population of Idaho and not limited to a particular age group or associated with an individual risk factor. The reasons for increases in reported incidence in Idaho might be multifactorial and involve factors not assessed in this study, such as expansion of >1 *emm* types and improvements in collection of diagnostic specimens by clinicians and passive reporting by laboratories. One limitation of our study is that we did not directly assess whether changes in the distribution of risk factors in the underlying population, such as the proportion of adults with skin injuries, could have led to increases in iGAS. For certain characteristics and risk factors, Idaho population data on estimated prevalence or proportion were unavailable (Appendix Table 2). For most factors, only minor changes over time were observed, and we did not identify any changes considered potentially responsible for the increase in iGAS incidence. Another limitation to the risk factor analysis was the small sample size during the baseline period—only 89 cases, some missing data. A third key limitation to our study was the lack of *emm* typing data from the 2008–2013 baseline period, because only 2% of cases had *emm* typing conducted, compared with 49% during 2014–2019. Therefore, we could not assess potential shifts in *emm* types, which might have contributed to increasing rates of iGAS. In Ireland, an increase in iGAS during 2012–2015 coincided with an upsurge in *emm3* (*34*). In the United States, expansion over time of select *emm* types, including 11, 77, and 92, was linked to increasing prevalence of antimicrobial drug–resistant iGAS infections (*35*). Factors that contribute to the emergence of *emm* types are not well understood but might include genetic changes in bacteria and acquisition of new virulence factors, as well as variations in population immunity (*30*). *emm59*, which emerged initially in Canada and more recently has been found in the southwestern United States, was the most common *emm* type in Idaho in 2014; all 5 cases were associated with outbreaks, but *emm59* declined during subsequent years (*19*,*36*).

In conclusion, in Idaho, iGAS is an urgent and consequential clinical and public health concern because of its severity, high case-fatality rate, and statewide increases. The absence of an identified risk factor contributing to increasing iGAS incidence in the general population suggests a potential role for vaccination as a preventive measure. Ongoing surveillance of state-level iGAS cases would help identify outbreaks, track regional trends in incidence, and monitor circulating *emm* types.

Appendix Additional information about a study of increasing incidence of invasive group A *Streptococcus* disease in Idaho, USA, during 2008–2019.
