# Supplementary material for: Increasing Incidence of Invasive Group A Streptococcus Disease, Idaho, USA, 2008–2019
Source: Emerg Infect Dis. 2022 Sep;28(9):1785–95. doi: 10.3201/eid2809.212129 (PMC9423907; doi:10.3201/eid2809.212129)
Supplement: Appendix — Additional information about a study of increasing incidence of invasive group A Streptococcus disease in Idaho, USA, during 2008–2019. [file 21-2129-Techapp-s1.pdf]

Article DOI: https://doi.org/10.3201/eid2809.212129

Increasing Incidence of Invasive Group A *Streptococcus* Disease, Idaho, USA, 2008–2019

Appendix

Additional Methods Information

Invasive Group A Streptococcus (iGAS) Incidence by Race and Ethnicity

Average annual incidence per 100,000 population for 2008–2019 was estimated for white non-Hispanic, Hispanic, and American Indian or Alaska Native persons using population estimates by race and ethnicity obtained from the Idaho Bureau of Vital Records and Health Statistics. For these estimates, the distribution of race and ethnicity in cases with missing data (n = 39) was assumed to be the same as that observed in cases with available data (n = 444). Average annual incidence was not estimated for persons of other races (Black, Asian, Native Hawaiian or Pacific Islander) because of the small number of cases.

Regression Analysis for Case-to-Case Comparison between Periods

Logistic regression models were used to compare demographics, underlying conditions, and other risk factors between the higher incidence period (2014–2019) and the baseline period (2008–2013) to identify any factors that might be positively associated with the higher incidence period. For 4 patients who had 2 cases of iGAS, only the first case was included in regression analyses. A multivariable logistic regression model included the following variables: age group, obesity, residence type, and injection drug use. These factors, which might explain the increase in iGAS cases, were chosen a priori based on the literature, with the assistance of a causal diagram to visualize relationships between variables, potential confounders, and the outcome (increase in iGAS) (*1–5*). Number of cases in each period was also considered when determining number of variables (degrees of freedom) in the multivariable model. Firth logistic regression was used to account for separation attributable to limited sample size and highly predictive risk factors (*6*).

References

<jrn>1. Factor SH, Levine OS, Schwartz B, Harrison LH, Farley MM, McGeer A, et al. Invasive group A streptococcal disease: risk factors for adults. Emerg Infect Dis. 2003;9:970–7. PubMed https://doi.org/10.3201/eid0908.020745</jrn>

<jrn>2. Langley G, Hao Y, Pondo T, Miller L, Petit S, Thomas A, et al. The impact of obesity and diabetes on the risk of disease and death due to invasive group A *Streptococcus* infections in adults. Clin Infect Dis. 2016;62:845–52. PubMed https://doi.org/10.1093/cid/civ1032</jrn>

<jrn>3. Steer AC, Lamagni T, Curtis N, Carapetis JR. Invasive group A streptococcal disease: epidemiology, pathogenesis and management. Drugs. 2012;72:1213–27. PubMed https://doi.org/10.2165/11634180-000000000-00000</jrn>

<unknown>4. Valenciano SJ, Onukwube J, Spiller MW, Thomas A, Como-Sabetti K, Schaffner W, et al. Invasive group A streptococcal infections among people who inject drugs and people experiencing homelessness in the United States, 2010–2017. Clin Infect Dis. 2021;73:e3718–26.</unknown>

<jrn>5. Greenland S, Pearl J, Robins JM. Causal diagrams for epidemiologic research. Epidemiology. 1999;10:37–48. PubMed https://doi.org/10.1097/00001648-199901000-00008</jrn>

<jrn>6. Heinze G, Schemper M. A solution to the problem of separation in logistic regression. Stat Med. 2002;21:2409–19. PubMed https://doi.org/10.1002/sim.1047</jrn>

Appendix Table 1. Invasive group A streptococcus *emm* types in Idaho, by year.

emm type	2012	2013	2014	2015	2016	2017	2018	2019	total
1	0	0	2	3	2	1	8	10	26
12	0	0	4	5	1	2	6	7	25
28	0	0	4	3	1	0	6	9	23
11	0	0	3	1	7	1	3	0	15
4	0	0	0	1	2	3	4	5	15
89	1	0	1	1	3	2	4	2	14
59	1	0	5	2	0	0	1	1	10
3.1	0	0	0	0	3	0	2	4	9
77	0	0	1	1	0	1	0	5	8
82	0	0	0	1	0	2	1	3	7
76.4	0	0	0	0	0	1	2	2	5
92	0	0	1	0	0	0	2	1	4
2	0	0	1	1	0	0	1	0	3
1.25	0	0	0	1	0	0	0	1	2
101	0	0	0	0	0	0	2	0	2
118	0	0	0	0	1	0	1	0	2
22	0	0	0	1	1	0	0	0	2
6.4	0	0	0	0	0	0	0	2	2
1.22	0	0	0	0	0	0	0	1	1
1.41	0	0	0	0	0	0	1	0	1
1.79	0	0	0	1	0	0	0	0	1
103	0	0	0	0	0	0	1	0	1
22.15	0	0	0	1	0	0	0	0	1
257	0	0	0	0	0	0	0	1	1
3.24	0	0	0	1	0	0	0	0	1
43.4	0	0	0	0	0	0	1	0	1
49	0	0	0	0	1	0	0	0	1
52.2	0	0	0	1	0	0	0	0	1
6	0	0	0	0	0	0	0	1	1
6.8	0	0	0	0	1	0	0	0	1
60.2	0	0	0	0	0	0	0	1	1
75	0	0	0	1	0	0	0	0	1
76	0	0	0	0	1	0	0	0	1
8	0	0	0	0	0	1	0	0	1
81	0	0	0	0	0	1	0	0	1
83.1	0	0	0	0	1	0	0	0	1
90.2	0	0	0	0	0	1	0	0	1
STG62647.0	0	0	0	0	0	0	0	1	1
								sum	194

Appendix Table 2. Idaho population estimates for selected demographic and risk factors.

Factor	Measurement	Data source	2008	2009	2010	2011	2012	2013	2014	2015	2016	2017	2018	2019
Total population	N	KFF[a]	1,484,300	1,507,700	1,538,900	1,547,300	1,562,500	1,580,900	1,598,800	1,617,200	1,650,700	1,682,100	1,719,600	1,750,900
American Indian/Alaska Native	N (% of total population)	KFF[a]	13,600 (0.9)	16, 900 (1.1)	15,300 (1.0)	18,700 (1.2)	14,500 (0.9)	15,800 (1.0)	14,600 (0.9)	18,000 (1.1)	18,900 (1.1)	17,500 (1.0)	18,100 (1.1)	15,900 (0.9)
White	N (% of total population)	KFF[a]	1,259,500 (84.9)	1,274,600 (84.5)	1,295,400 (84.2)	1,297,600 (83.9)	1,305,400 (83.5)	1,317,000 (83.3)	1,325,000 (82.9)	1,339,100 (82.8)	1,362.500 (82.5)	1,381,400 (82.1)	1,407,600 (81.9)	1,429,500 (81.6)
Hispanic/ Latino	N (% of total population)	KFF[a]	154, 600 (10.4)	161, 100 (10.7)	172,700 (11.2)	175,200 (11.3)	180,000 (11.5)	186,300 (11.8)	193,400 (12.1)	192,800 (11.9)	202,200 (12.2)	207,700 (12.3)	217,500 (12.6)	225,600 (12.9)
Obesity	N (% of adults)	Idaho BRFSS[b]	261,664 (25.2)	273,573 (25.1)	294,677 (26.9)	296,226 (27.0)	294,722 (26.8)	330,015 (29.6)	326,369 (28.9)	322,889 (28.6)	318,290 (27.4)	353,688 (29.3)	339,271 (28.4)	353,047 (29.4)
Diabetes	N (% of adults)	Idaho BRFSS[b]	76,274 (7.0)	90,107 (8.0)	90,907 (7.9)	107,580 (9.4)	98,680 (8.5)	98,977 (8.4)	91,799 (7.6)	99,277 (8.1)	111,490 (8.9)	111,476 (8.7)	132,857 (10.2)	137,374 (10.3)
Coronary heart disease or angina	N (% of adults)	Idaho BRFSS[b]	42,915 (4.0)	43,276 (3.9)	40,662 (3.6)	47,131 (4.1)	41,809 (3.6)	42,664 (3.6)	40,324 (3.4)	37,998 (3.1)	48,854 (3.9)	41,900 (3.3)	47,581 (3.6)	50, 141 (3.8)
HIV	Prevalence per 100,000	NCHHSTP[c]	56.6	58.9	67.5	69.8	73	75.6	74.2	75.4	78.2	80.2	81.9	84
End-stage renal disease	Prevalent count	USRDS[d]	n/a	1,845	1,983	1,999	2,081	2,153	2,256	2,359	2,438	2,521	2,569	n/a
Cancer, all types	10 year limited-duration prevalence	CDRI[e]	34,412	35,852	37,183	38,303	39,336	40,455	41,259	42,228	43,302	44,373	45,444	46,644
Nursing home residents	Total	KFF[f]	4,537	4,422	4,358	4,460	4,137	3,900	3,901	3,728	3,921	3,319	n/a	3,286
Homeless	Total	HUD[g]	1,464	1,939	2,346	2,199	1,968	1,781	2,104	1,966	2,347	2,037	2,012	2,315

[a]Kaiser Family Foundation, https://www.kff.org/other/state-indicator/distribution-by-raceethnicity/
[b]Idaho Behavioral Risk Factor Surveillance System, https://www.gethealthy.dhw.idaho.gov/idaho-brfss. Due to changes in methods, data from 2011 and later are not directly comparable with data from 2010 and earlier.
[c]National Center for HIV, Viral Hepatitis, STD, and TB Prevention AtlasPlus, https://gis.cdc.gov/grasp/nchhstpatlas/tables.html
[d]United States Renal Data System, https://usrds.org/data-query-tools/esrd-prevalent-count/
[e]Cancer Data Registry of Idaho
[f]Kaiser Family Foundation, https://www.kff.org/other/state-indicator/number-of-nursing-facility-residents/
[g]United States Department of Housing and Urban Development, https://www.hudexchange.info/programs/coc/coc-homeless-populations-and-subpopulations-reports/

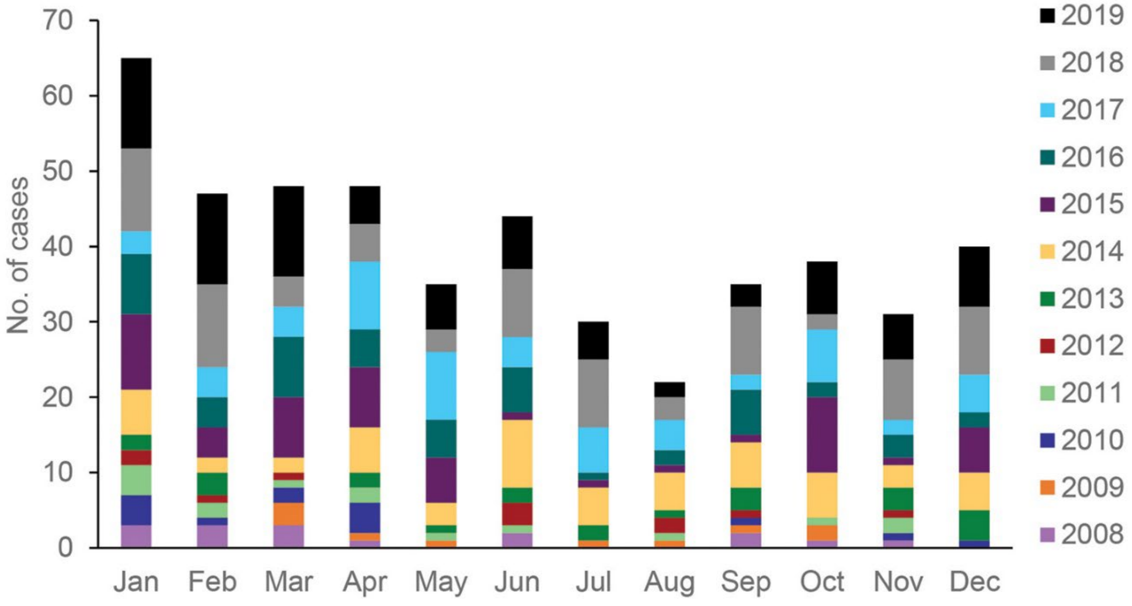

Appendix Figure 1. Variation by month in invasive group A streptococcus incidence from an investigation in Idaho comparing cases reported during 2014–2019 with cases from a lower-incidence baseline period, 2008–2013.

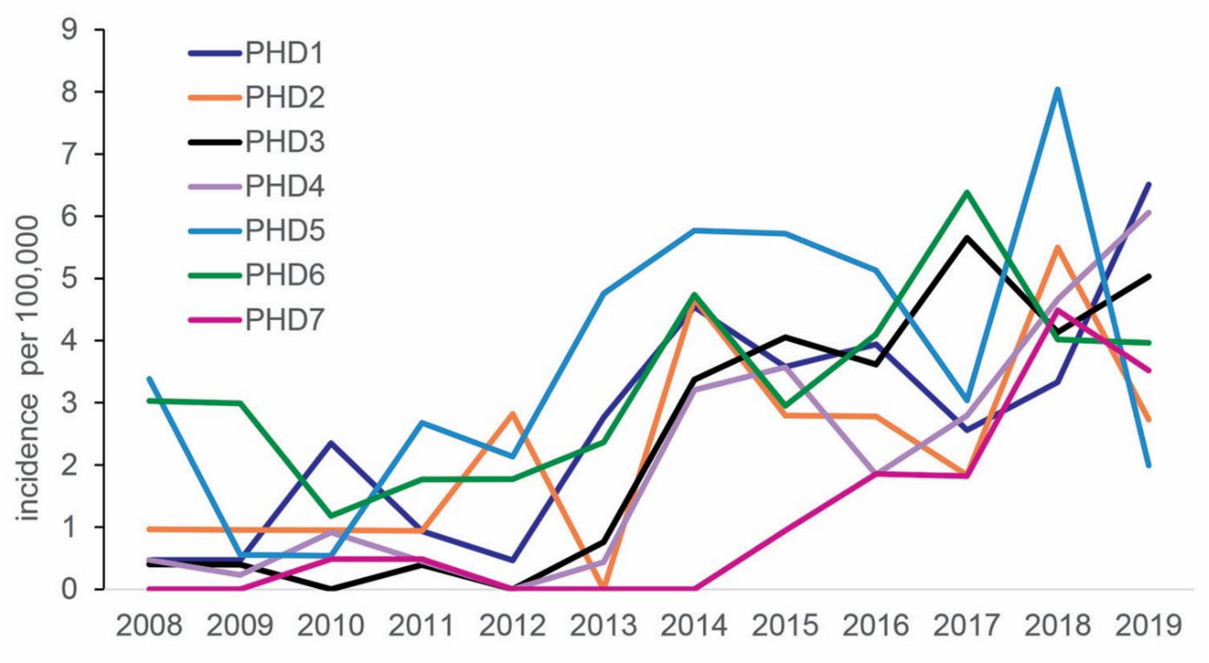

Appendix Figure 2. Variation by public health district in invasive group A streptococcus incidence from an investigation in Idaho comparing cases reported during 2014–2019 with cases from a lower-incidence baseline period, 2008–2013. PHD, public health district.
